# Baseline Characteristics of the TOPaZ Study: Randomised Trial of Teriparatide and Zoledronic Acid Compared with Standard Care in Adults with Osteogenesis Imperfecta

**DOI:** 10.1007/s00223-025-01440-3

**Published:** 2025-11-08

**Authors:** Jannie Dahl Hald, Christopher Weir, Catriona Keerie, Lorna Dewar, Morag MacLean, Lynsey Milne, Richard Keen, Jennifer Walsh, Kenneth Poole, Bente Langdahl, John R. Lindsay, Nazim Ghouri, Rosemary J Hollick, Terry Aspray, Rachel K. Crowley, Martine Cohen-Solal, Zaki Hassan-Smith, Stephen Tuck, Elizabeth Curtis, Nick Harvey, E. Marelise W. Eekhoff, Johannes Feenstra, Geeta Hampson, Mike Stone, Jane Turton, Prashanth Patel, Mashood Siddiqi, Robin Munro, Matthew Roy, Zoe Paskins, Deepa Narayanan, Ellen Malcolm, Muhammad Kassim Javaid, Patricia Osborne, Jonathan C. Y. Tang, Wayne Lam, David Moore, Holly A. Black, Andrew D. Duckworth, Navnit Makaram, Tianyu Guo, Gregor Stenhouse, Stuart H. Ralston

**Affiliations:** 1 Department of Endocrinology and Internal Medicine, Aarhus University Hospital, Aarhus, Denmark; 2 Centre for Rare Disorders, Aarhus University Hospital, Aarhus, Denmark; 3 Edinburgh Clinical Trials Unit, Usher Institute, University of Edinburgh, Edinburgh, UK; 4 Metabolic Bone Clinic, Royal National Orthopaedic Hospital NHS Trust, London, UK; 5 Metabolic Bone Centre, University of Sheffield, Sheffield, UK; 6 Cambridge University Hospitals NHS Trust, Addenbrookes Hospital, Cambridge, UK; 7 Belfast Health and Social Care Trust, Royal Victoria Hospital, and Musgrave Park Hospital, Belfast, UK; 8 Endocrinology, Queen Elizabeth University Hospital, University of Glasgow, Glasgow, UK; 9 Aberdeen Centre for Arthritis and Musculoskeletal Health (Epidemiology), University of Aberdeen, Aberdeen, UK; 10 Bone Clinic- Freeman Hospital, University of Newcastle, Newcastle, UK; 11 Department of Endocrinology, Saint Vincent’s University Hospital, Dublin, Ireland; 12 Rare Disease Clinical Trial Network, University College Dublin, Dublin, Ireland; 13 Department of Rheumatology, Inserm U1132 Bioscar, F-75010 Paris, France; 14 Aston Medical School, Aston University, Birmingham, UK; 15 Department of Endocrinology, Queen Elizabeth Hospital, Birmingham, UK; 16 South Tees Hospitals NHS Foundation Trust, Rheumatology, Middlesborough UK; 17 MRC Lifecourse Epidemiology Centre, University Hospital Southampton, Southampton, UK; 18 Center for Rare Bone Diseases, Department of Endocrinology, Amsterdam Reproduction and Development Research Institute, Amsterdam, Netherlands; 19 Isala Hospital Zwolle, Zwolle, Netherlands; 20 Clinical Chemistry and Metabolic Medicine, Guy’s and St Thomas NHS Trust, London, UK; 21 Metabolic Bone Clinic, University Hospital Llandough, Cardiff, UK; 22 Chemical Pathology and Metabolic Disease, University Hospitals of Leicester NHS Trust, Leicester, UK; 23 Metabolic Bone Diseases, University Hospitals of Liverpool Group, Liverpool, UK; 24 University Hospital Wishaw, Wishaw, UK; 25 University Hospitals Bristol NHS Trust, Bristol Royal Infirmary, Bristol, UK; 26 University of Keele, Rheumatology, Stoke-on-Trent UK; 27 The Centre for Metabolic Bone Disease, Hull University Teaching Hospitals NHS Trust, Hull, UK; 28 Department of Medicine, Ninewells Hospital and Medical School, Dundee, UK; 29 Nuffield Orthopaedic Centre, University of Oxford, Oxford, UK; 30 Brittle Bone Society, Dundee, UK; 31 Bioanalytical Facility, Norwich Medical School, Norwich, UK; 32 South East Scotland NHS Genetics Service, Edinburgh, UK; 33 Department of Orthopaedic Surgery, Royal Infirmary of Edinburgh, NHS Lothian, Edinburgh, UK; 34 Centre for Population Health Sciences, Usher Institute, University of Edinburgh, Edinburgh, UK; 35 Department of Radiology, Royal Infirmary of Edinburgh, NHS Lothian, Edinburgh, UK; 36 Department of Rheumatology, Western General Hospital, NHS Lothian, Edinburgh, UK; 37 Institute of Genetics and Cancer, Western General Hospital, University of Edinburgh, Edinburgh, UK; 38 NIHR Southampton Biomedical Research Centre, University of Southampton and University Hospital NHS Foundation Trust, Southampton, UK

**Keywords:** Osteogenesis imperfecta, Genetic, Bisphosphonate, Teriparatide, Clinical trial

## Abstract

**Introduction:**

Osteogenesis imperfecta (OI) is a rare disorder causing multiple fractures throughout life. No treatment has been shown to reduce the risk of fractures in OI. Here, we present the baseline characteristics of participants in the Treatment of Osteogenesis Imperfecta with Parathyroid Hormone and Zoledronic Acid (TOPaZ) trial. The aim of the trial is to determine whether teriparatide and zoledronic acid are superior to standard care in reducing the risk of clinical fractures.

**Methods:**

We summarised data on the baseline characteristics of TOPaZ participants, including demographics, genetic diagnosis, clinical features, bone density measurements, previous treatments, and fracture history.

**Results:**

We recruited 350 adults with a clinical diagnosis of OI in 27 European referral centres between June 2017 and October 2022. Overall, 266 (76.2%) had type I OI, 55 (15.8%) had type IV, and 19 (5.4%) had type III. The type was unknown in 9 (2.6%). Blue sclera were noted in 80.8%, and 35.8% had dentinogenesis imperfecta. Bisphosphonates had been administered to 28.1% in the 2 years prior to enrolment. Pathogenic variants in *COL1A1* or *COL1A2* were found in 87.6%. Fractures occurring in the 2 years prior to enrolment were not associated with bone density.

**Conclusions:**

The TOPaZ population represents a unique cohort with which to study the genetic epidemiology and outcome of OI in relation to bone density and biochemical markers of bone turnover. When the trial reports, it will also provide new insights into the effect of an anabolic therapy, followed by antiresorptive treatment in the management of OI.

## Introduction

Osteogenesis imperfecta (OI) is a rare connective tissue disorder causing multiple fractures throughout life. The main impact of the disease is on the skeleton, and population-based studies indicate that there is an eightfold increase in fractures in people with OI as compared with population-based controls [1, 2]. This is most marked during childhood but persists throughout life [2]. Osteogenesis imperfecta is also associated with impaired muscle function and strength and is often associated with immobility [3] which further increases the risk of fracture. Major advances have been made in understanding the pathogenesis of OI over recent years. At the present time, more than 20 subtypes of OI have been described due to pathogenic variants in genes that influence production, modification, folding, and crosslinking of type I collagen, as well as bone mineralisation and osteoblast function or differentiation [4]. In addition to causing increased bone fragility, OI is associated with an increased risk of cardiovascular and respiratory disease [5] craniofacial disease [6], gastrointestinal problems [5] and osteoarthritis [7] as well as problems with tendons and ligaments [1]. Pain and fatigue are common symptoms, but the underlying causes are not well understood [8]. There is no licensed medical treatment for osteogenesis imperfecta, but intravenous and oral bisphosphonates are widely used in clinical practice for the treatment of OI in adults and children. There is good evidence that these drugs increase bone mineral density (BMD) versus placebo, but they have not been shown to prevent fractures [9, 10]. This observation illustrates that OI is quite distinct from osteoporosis, even though both conditions are associated with an increased risk of fractures. There have been relatively few randomised controlled trials of medical treatment in OI, but most of these have focused on medicines that increase bone mineral density. One of these is teriparatide, which has been evaluated in the treatment of adults with OI and has been found to increase BMD compared with placebo [11], but this study was not powered to look at fracture risk [11]. Another medicine under development is setrusumab, which is a monoclonal antibody directed against sclerostin. The dose-ranging phase 2b Asteroid study of setrusumab has been completed, and positive effects on bone density were demonstrated over 12 months, but this was a small study, and no differences in annualized fracture rates between different doses were observed [12].

With this background, we have designed the Treatment of Osteogenesis Imperfecta with Parathyroid Hormone and Zoledronic Acid (TOPaZ) trial to determine if the bone anabolic medicine teriparatide, followed by the bisphosphonate zoledronic acid to maintain increases in BMD that might occur, would be beneficial in reducing the risk of clinical fractures in adults with OI. We have previously published the protocol of this study [13] and here, we present the baseline characteristics of the participants enrolled in TOPaZ. As well as describing the participant characteristics, we present an analysis of the relation between Sillence type and BMD, the proportion of individuals with normal BMD, osteopenia, and osteoporosis, and the relation between type of pathogenic variant, bone density, and bone turnover markers. The TOPaZ trial is the first trial in which this combination of agents has been used and, to our knowledge, is the first trial that has been designed in which clinical fractures are a primary outcome. Due to the large number of participants, this trial will provide data on fracture prevention with these agents, as well as clinical and genetic data in a large, well-characterized cohort of adults with OI.

## Patients and Methods

### Study Design

The TOPAZ trial (Registration number ISRCTN15313991) is a multicenter open-label randomised parallel-group trial that was designed to determine if a 2-year course of bone anabolic therapy with TPTD followed by a single infusion of ZA to maintain the increase in BMD is superior to standard care at reducing the risk of new clinical fractures in adults with OI. The design of the TOPAZ trial has previously been reported [13]. Treatment of participants allocated to standard care was left to the discretion of the local investigator and could involve antiresorptive drugs like bisphosphonates or denosumab, calcium, and Vitamin D supplements, or Vitamin D supplements alone, or no bone-targeted treatment. Bone anabolic drugs like teriparatide, romosozumab, and investigational drugs were prohibited in the standard care arm.

### Recruitment

Potential participants at each study centre were approached directly as they attended routine outpatient clinic visits or by telephone or letter. Participants were invited to take part in the TOPaZ trial if they had a clinical diagnosis of OI and did not have a contraindication to the use of ZA or TPTD. Written informed consent was obtained from all participants by the local principal investigator or a delegated member of the study team. The inclusion and exclusion criteria for participation in the trial have been published previously [13]. Key inclusion criteria were those aged 18 and over with a clinical diagnosis of OI. Participants with a contraindication to ZA or TPTD were excluded. Enrolment into the study took place at 27 centres in five European countries between June 2017 and February 2023. The last patient, last visit was on February 22nd 2025, and the study end date was 31st July 2025. The results are expected to be available before the end of 2025.

### Clinical Assessments

We recorded height, weight, and sex. Local investigators were asked to examine patients and note the presence of blue sclera, dentinogenesis imperfecta, bone deformity, and use of a hearing aid for deafness. Comorbidities, alcohol intake, and smoking habits were recorded by interview coupled with an evaluation of the participants’ health record. Participants were asked to report fractures in the previous 2 years and fractures throughout life. We also recorded use of bone-targeted treatment in the two years prior to enrolment and throughout life.

### Genetic Testing

All participants were offered genetic testing as part of the protocol. This was achieved by targeted exome sequencing in a panel of 16 genes previously associated with OI [13]. All pathogenic variants were documented by Sanger sequencing. Pathogenicity was determined by ACMG criteria [14]. Pathogenic variants were further classified into three subgroups: those that were expected to result in haploinsufficiency due to nonsense-mediated RNA degradation (quantitative variants); those expected to result in a change to the protein sequence (qualitative variants); and those affecting a splice site where the consequences on protein structure and RNA degradation were unclear (splice site variants).

### Biochemical Measurements

Serum creatinine, serum calcium, serum total alkaline phosphatase, and albumin were analysed at participating centres by the local laboratories according to standard techniques. Estimated glomerular filtration rate (eGFR) was calculated from serum creatinine, age, and weight using the Cockcroft-Gault equation. We did not implement cross-calibration of these bloods between centres as they were primarily performed as safety assessments.

Measurements of PINP and CTX were performed at the bioanalytical facility at the University of East Anglia using an Electrochemiluminescence immunoassay (ECLIA) on a Cobas e601 analyser (Roche Diagnostics, Germany). The inter-assay coefficient of variation (CV) for CTX was ≤ 3% between the measuring range 0.01 and 6.00 µg/L with the sensitivity of 0.01 µg/L. The reference range was 0.16–0.85 µg/L. The PINP inter-assay CV was ≤ 3% between the assay range of 5–1200 µg/L with the sensitivity of 5 µg/L. The reference range was 20–76 µg/L. The inter-assay coefficient of variation (CV) for CTX was ≤ 3% between 0.2 and 1.5 µg/L with the sensitivity of 0.01 µg/L. The reference range was 0.16–85 µg/L.

### Bone Mineral Density

Bone mineral density (BMD) was measured by dual x-ray energy absorptiometry (DXA) at the spine and hip according to standard techniques at the participating centres. In 38%, the assessments were done using Hologic Discovery machines; in 27%, Hologic Horizon machines were used, and in 35%, Lunar machines were used. Data were expressed as g/cm^2^ and as T-scores, which were generated based on the manufacturer’s reference ranges. Repeat BMD measurements in individual participants were performed on the same densitometer. We did not attempt to cross-calibrate the BMD results across centres since the primary endpoint of the study was clinical fracture, and the reason for performing BMD measurements was to evaluate the effects of the interventions on BMD as an explanatory variable within patients rather than to compare changes in BMD between centres.

In several participants, BMD measurements were not feasible for technical reasons at specific sites. For measurements at the hip, the most common reason for missing data was metalwork in place because of previous surgery. For measurements at the spine, the most common reasons for missing data include metalwork in place as well as pre-existing vertebral compression fractures in the lumbar spine, and co-existing osteoarthritis or degenerative disk disease at the lumbar spine. The decision to exclude a site for BMD measurement in individual participants was left to the discretion of radiographers at the participating centres according to their local standard operating procedures.

### Vertebral Fractures

The presence and severity of vertebral fractures was evaluated on lateral spine x-rays which were obtained at baseline and the end of study. Anonymized images were uploaded to the study database and evaluated by two expert musculoskeletal radiologists blinded to treatment allocation who recorded the site and severity of vertebral fractures using the Genant semiquantitative method [15]. The categories used were mild (20–25% height loss), moderate (25–40% height loss), and severe (> 40% height loss). These assessments were done using vertebral height measurements. We did not routinely perform double evaluations. If an adjudicator was uncertain about the presence or severity of a vertebral fracture, they were instructed to seek a second opinion from the other adjudicator and reach a consensus. However, in practice, this was not required for any of the adjudications performed in the study.

### Statistical Analysis

Statistical analysis was performed using SPSS version 29. Differences between groups for continuous variables were assessed by ANOVA for normally distributed data or the Mann–Whitney test for data that were not normally distributed. We used the general linear model ANOVA to analyse differences between biochemical markers of bone turnover and bone density in relation to the type of pathogenic variant, with the addition of recent bisphosphonate treatment into the model. For this analysis, differences between groups were evaluated by post-hoc testing.

## Results

We recruited 350 participants to the trial from 27 referral centres located in five different countries, but one withdrew from the study, requesting no further contact or use of their data, leaving a final sample size of 349 subjects where data were available for analysis. The distribution was UK (n = 303, 86.5%), Republic of Ireland (n = 10, 2.9%), The Netherlands  (n = 9, 2.6%), France (n = 10, 2.9%), and Denmark (n = 18, 5.2%). Relevant clinical characteristics of the study population are shown in Table 1. The average age was 43.7 years (range 18–84 years). The average height was 156 cm (range 75–187 cm), average weight was 69.7 kg (range 17–160 kg), and average BMI was 28.7 (range 17–79). In total, 188 (53.9%) of participants were female, and of these women, 73 (38.8%) were postmenopausal. In 266 (76.2%), the Sillence Classification was type I osteogenesis imperfecta; in 55 (15.8%), type IV; and 19 (5.4%), type III. The type was coded as unknown in 9 (2.6%). On physical examination, 282 (80.8%) had blue sclera, 125 (35.8%) had dentinogenesis imperfecta; 68 (27.3%) used a hearing aid for deafness, and 220 (63.0%) were considered to have bone deformity. Measurements of BMD were possible at the spine in 322 individuals (92.3%), at the femoral neck in 285 (81.7%), and at the total hip in 284 (81.4%). In the remainder, measurements were not feasible due to metalwork and other image artefacts that prevented an accurate assessment of BMD. The BMD T-scores were lowest at the lumbar spine, with an average T-score value of -2.15, followed by femoral neck (T score = − 1.48) and total hip (T-score = − 1.23).

**Table 1:** Clinical, genetic and biochemical characteristics of Study Population

Variable	Value
Number of participants	349
Age	43.7 ± 13.8
Female sex	188 (53.9%)
Height (cm)	156 ± 17.0
Weight (Kg)	69.7 ± 19.3
Body Mass Index (Kg/M^2^)	28.7 ± 7.1
Current smoker^1^	58 (23.3%)
Alcohol use^1^	144 (57.8%)
*Sillence classification*	
Type I	266 (76.2%)
Type III	19 (5.4%)
Type IV	55 (15.8%)
Not classified	9 (2%)
*Clinical examination*	
Blue Sclera	282 (80.8%)
Dentinogenesis imperfecta	125 (35.8%)
Hearing aid use^1^	68 (27.3%)
Bone Deformity	220 (63.0%)
*Genetic analysis*	
Pathogenic variant *COL1A1*	218 (62.6%)
Pathogenic variant *COL1A2*	83 (23.9%)
Pathogenic variant other gene^2^	9 (2.5%)
Variant of uncertain significance	7 (2.0%)
No pathogenic variant detected	31 (8.9%)
*Bone Mineral Density*	
Lumbar spine BMD (g/cm^2^)	0.856 ± 0.187
Lumbar spine T-score	− 2.15 ± 1.76
Femoral neck BMD (g/cm^2^)	0.741 ± 0.148
Femoral neck T-score	− 1.48 ± 1.26
Total hip BMD (g/cm^2^)	0.827 ± 0.145
Total hip T-score	− 1.23 ± 1.24
*Participants with previous fractures*	
Within past 2 years	163 (46.7%)
More than 2 years ago	333 (95.4%)
Participants with ≥ 1 vertebral fracture at baseline	177 (51.0%)
*Biochemistry*	
eGFR ml/min	118 ± 45.7
Serum Alkaline phosphatase U/L	83.0 ± 26.0
Serum PINP µg/L	32.2 [7.9-190.2]
Serum CTX µg/L	0.18 [0.01–0.83]

Values are mean ± SD, numbers (%), or median [range] for fractures serum PINP and serum CTX

^1^Data on alcohol smoking and hearing aid use were available on 249 participants

^2^Pathogenic variants were also found *FKBP10* (n = 3), *IFITM5* (n = 3), *P3H1* (n = 1), *SERPINF1* (n = 1), and *BMP1* (n = 1). Reference ranges for the biochemical analytes are shown in the methods section

### Genetic Testing

The results of genetic testing were available on 348 individuals. Pathogenic variants in *COL1A1* were detected in 221 (63.3%) and in *COL1A2* in 85 (24.4%). Pathogenic variants in *FKBP10* were detected in 3 (0.9%), *IFITM5* in 3 (0.9%), *P3H1* in 1 (0.3%), *PLS3* in 1 (0.3%), *SERPINF1* in 1 (0.3%), and *BMP1* in 1 (0.3%)*.* One individual had a pathogenic variant in *CRTAP* but in heterozygous form. This was considered a variant of uncertain significance, given that OI secondary to *CRTAP* variants is a recessive condition with biallelic pathogenic variants.

### Previous Fractures

**Fig. 1:**
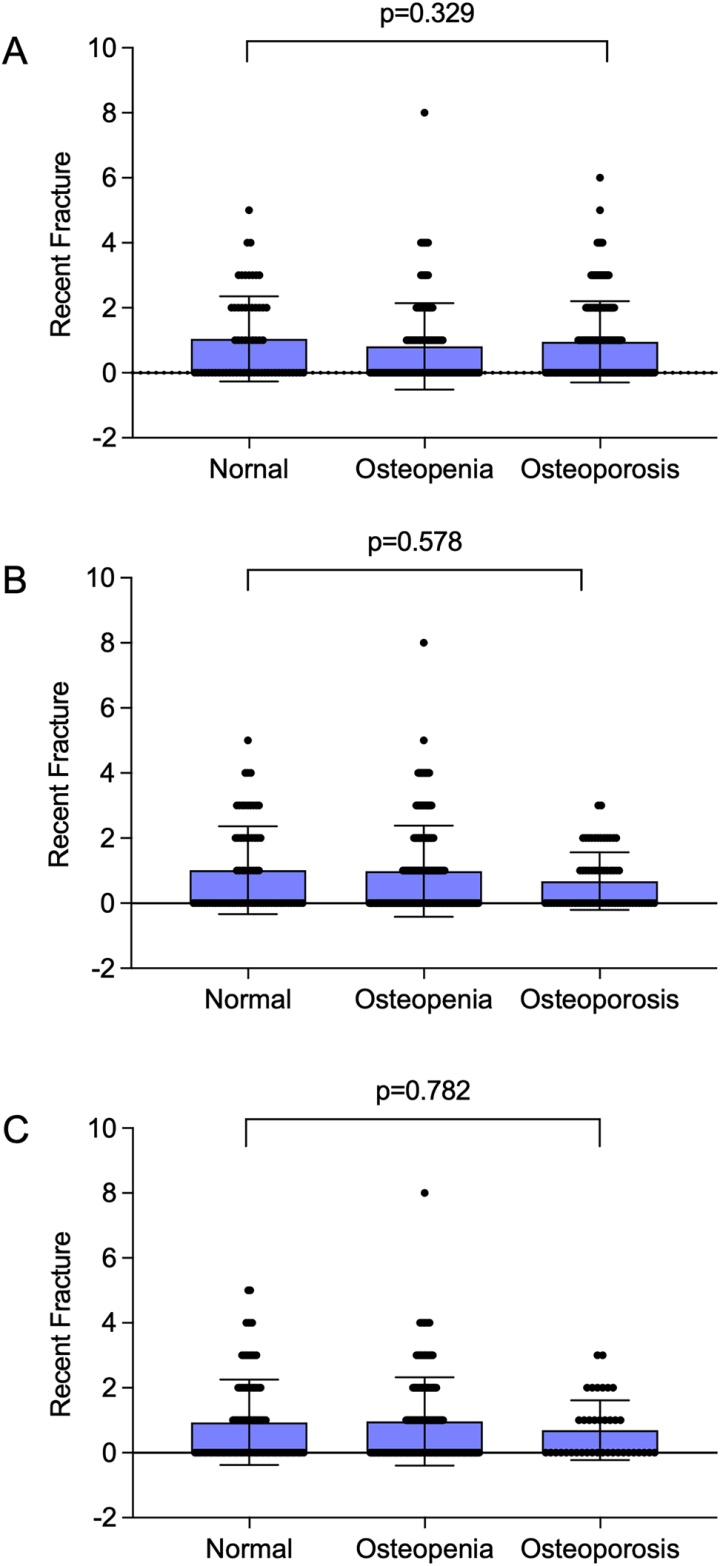
Recent fractures in relation to the presence of normal BMD osteopenia, and osteoporosis at baseline. Columns are means and standard deviations with individual values shown as circles. Panel A, Lumbar spine BMD; panel B, Femoral Neck BMD, Panel C Total Hip BMD. There was no significant difference between recent fracture numbers in categories of patients with normal BMD, osteopenia and osteoporosis at any skeletal site

Information on previous self-reported fractures was available in all 349 individuals. The median number of fractures per patient in the two years prior to enrolment was 0 [range 0–8], but prior to this, the median per patient number of fractures was 11 [range 0–257]. A total of 2571 self-reported fractures occurred in the study cohort overall. Of these, 831 (32.3%) were reported to have occurred at age less than 10 years; 866 (33.7%) were reported to have occurred between 10 and 20 years of age; and 789 (30.7%) were reported to have occurred above the age of 20 years. The time of fracture was not recorded in 85 instances (3.3%). Radiologically confirmed vertebral fractures were present at baseline in 177 (51.0%) individuals. In 156 individuals, fractures were in the thoracic spine, and in 95 individuals, they were in the lumbar spine. There was no significant difference in the number of participants with fractures in the two years prior to enrollment in subjects with normal BMD, osteopenia and osteoporosis (Figure 1).

### Comorbidities

Data on comorbidities were available in 249 subjects. Asthma was present in 50 (20.1%) and COPD in 5 (2.0%). There was a history of cardiovascular disease in 32 (12.9%), cerebrovascular disease in 7 (2.8%), and peripheral vascular disease in 1 (0.4%). Osteoarthritis of the spine was present in 39 (15.7%), of the hips in 39 (15.7%), of the knees in 49 (19.7%), and of the hands in 35 (14.1%). There was a previous diagnosis of psoriasis in 10 (4%) and of eczema in 23 (9.2%), and a history of cancer in 11 (4.4%). There was a previous diagnosis of epilepsy in 13 (5.2%). One individual (0.4%) had a diagnosis of polymyalgia rheumatica, 2 (0.8%) had a diagnosis of rheumatoid arthritis, and 2 (0.8%) had a diagnosis of ankylosing spondylitis. Hypothyroidism was reported in 18 participants (7.2%).

### Previous Bone Targeted Treatment

Previous use of bone targeted treatments is summarised in Table 2. Overall, 98 individuals (28.1%) had received any bisphosphonate at randomization or in the 2 years prior to randomization of which 34 (9.7%) had used ZA, followed by alendronic acid in 31 (8.9%), risedronate in 18 (15.2%); ibandronate in 2 (0.6%) and pamidronate in 17 (4.9%). Other bone targeted medications used in the 2 years prior to enrollment included calcium and Vitamin D supplements in 59 (16.9%) Vitamin D supplements in 152 (43.6%) denosumab in 3 (0.9%) strontium ranelate in 1 (0.3%), and teriparatide in 1 (0.3%) Overall, 245 (70.2%) of participants had been treated with bisphosphonates in the past. The most commonly used were alendronic acid in 97 (27.8%), pamidronate in 80 (22.9%), risedronate in 68 (19.5%), and zoledronic acid in 75 (21.5%). Other medicines used in in the past, included calcium and Vitamin D supplements in 69 (19.8%), Vitamin D supplements in 163 (46.7%) teriparatide in 8 (2.3%), denosumab in 5 (1.4%) strontium ranelate in 4 (1.1%), and calcitonin in 3 (0.9%).

**Table 2:** Use of bone targeted medicines in study population

Drug	Within previous two years	More than two years previously
Bisphosphonates	98 (28.1%)	245 (70.2%)
*Zoledronic acid*	*34 (9.7%)*	*75 (21.5%)*
*Alendronic acid*	*31 (8.9%)*	*97 (27.8%)*
*Risedronate*	*18 (5.2%)*	*68 (19.5%)*
*Ibandronate*	*2 (0.6%)*	*11 (3.2%)*
*Etidronate*	*1 (0.3%)*	*1 (0.3%)*
*Pamidronate*	*17 (4.9%)*	*80 (22.9.%)*
Non bisphosphonates	173 (49.6%)	190 (54.4%)
*Calcium and vitamin D supplements*	*59 (16.9%)*	*69 (19.8%)*
*Vitamin D supplements*	*152 (43.6%)*	*163 (46.7%)*
*Teriparatide*	*1 (0.3%)*	*8 (2.3%)*
*Denosumab*	*3 (0.9%)*	*5 (1.4%)*
*Strontium ranelate*	*1 (0.3%)*	*4 (1.1%)*
*Calcitonin*	*0 (0%)*	*3 (0.9%)*

Recent use refers to use of the medication at the time of randomization or within the previous two years. The numbers add up to more than 100% since participants may have received more than one medication

### Association Between Osteogenesis Imperfecta Subtype and Bone Density

Analysis of associations between Sillence subtypes and the presence of normal BMD, osteopenia, and osteoporosis is shown in Table 3. Pearson’s Chi-Square analysis showed that there was a significant overall difference between Sillence subtypes and the proportion of individuals with normal BMD, osteopenia, and osteoporosis at the lumbar spine, femoral neck, and total hip, but interpretation of this data was limited by the fact that the number of observations in several cells was well below that of the expected counts and because BMD data were missing due to technical factors in up to 18.2% of participants. When data from all individuals were combined, normal BMD values at the lumbar spine were recorded in 62 (17.8%); osteopenia in 103 (29.5%); and osteoporosis in 157 (45.0%) with missing data in 27 (7.7%). At the femoral neck, normal BMD values were recorded in 72 (20.6%), osteopenia in 148 (42.4%), and osteoporosis in 65 (18.6%) with missing data in 64 (18.3%). At the total hip, normal BMD values were recorded in 97 (27.7%), osteopenia in 148 (42.4%), and osteoporosis in 39 (11.2%) with missing data in 65 (18.6%). We also examined possible associations between BMD values in g/cm^2^ in the different Sillence subtypes. This data is shown in Table 4. Lumbar spine BMD was significantly higher in type I OI versus types III and IV, spine BMD in other types was higher than type IV, and spine BMD was significantly higher in type IV OI versus type III. For the femoral neck site, BMD in type I was higher than in type IV and femoral neck BMD in other types was significantly higher than both type III and IV. For the total hip site, there was no significant difference in BMD values between the groups.

**Table 3:** Proportions of individuals with normal BMD, osteopenia and osteoporosis based on T-score values according to Sillence type

	OI Type I (n=266)	OI Type IV (n=55)	OI Type III (n=19)	Other (n=9)
*Lumbar spine*				
Normal	54 (20.3%)	7 (12.7%)	0 (0%)	1 (11.1%)
Osteopenia	91 (34.2%)	10 (18.2%)	1 (5.3%)	1 (11.1%)
Osteoporosis	111 (41.7%)	31 (56.4%)	10 (52.6%)	5 (55.6%)
Missing	10 (3.8%)	7 (12.7%)	8 (42.1%)	2 (22.2%)
*Femoral Neck*				
Normal	65 (24.4%)	4 (7.3%)	0 (0%)	3 (33.3%)
Osteopenia	131 (49.2%)	13 (23.6%)	0 (0%)	4 (44.4%)
Osteoporosis	52 (19.5%)	10 (18.2%)	3 (15.8%)	0 (0%)
Missing	18 (6.8%)	28 (50.9%)	16 (84.2%)	2 (22.2%)
*Total*				
Total Hip				
Normal	87 (32.7%)	8 (14.5%)	0 (0%)	2 (22.2%)
Osteopenia	130 (48.9%)	12 (21.8%)	1 (5.3%)	5 (55.6%)
Osteoporosis	30 (11.3%)	7 (12.7%)	2 (10.5%)	0 (0%)
Missing	19 (7.1%)	28 (50.9%)	16 (84.2%)	2 (22.2%)

Normal BMD = T score > 1.0; osteopenia T-score > -1.0 to -2.5; osteoporosis T-score > -2.5. Values are numbers and column (%). At the lumbar spine, the Pearson Chi square statistic was 55.8, with 9 degrees of freedom giving a *p*-value of < 0.001. However, 7 cells (43.8%) had expected counts less than 5 compared with a minimum expected count of 0.7. Corresponding values for the femoral neck were 124.9 (*p* < 0.001) but 7 cells (43.8%) had expected counts less than 5 with a minimum expected count of 1.65. Corresponding values for the total hip were 120.5 (*p* < 0.001) but 6 cells (37.5%) had expected counts less than 5 with a minimum expected count of 1.01.

**Table 4:** Bone mineral density at spine and hip in relation to Sillence Type

	OI type I	OI type IV	OI type III	Other
Lumbar spine	0.876 ± 0.183^a^	0.801 ± 0.148^b^	0.583 ± 0.184	0.845 ± 0.204^c^
Femoral Neck	0.747 ± 0.146^d^	0.658 ± 0.115	0.530 ± 0.049	0.867 ± 0.153^e^
Total Hip	0.834 ± 0.144	0.780 ± 0.128	0.600 ± 0.130	0.839 ± 0.173

Values are mean ± standard deviation BMD in g/cm^2^. Significance between groups indicated by:

^a^*p* < 0.001 from type III, *p* = 0.046 from type IV, ^b^*p* =0.003 from type III; ^c^*p* = 0.019 from type III, ^d^*p* = 0.012 from type IV; ^e^*p* = 0.022 from type III and *p* =0.004 from type IV. For lumbar spine BMD measurements were available in 260 individuals with type I, 49 with type IV, 10 with type III and 7 for other types. For femoral neck, corresponding values were 251 (Type I), 28 (IV), 2 (III) and 7 (other). For total hip corresponding values were 250 (Type I), 28 (IV) 2 (Type III) and 7 (other)

### Effect of Recent Bisphosphonate Treatment on BMD and Biochemical Markers of Bone Turnover

The effects of recent bisphosphonate treatment on bone density and biochemical markers of bone turnover are shown in Table 5. Serum CTX values and PINP values were significantly lower in those who had received bisphosphonates in the past 2 years as compared with those who had not received bisphosphonates. There were no significant differences in BMD values, expressed as g/cm^2^ and T-score at the spine according to whether or not bisphosphonate treatment had been given in the previous 2 years. However, femoral neck T-score, total hip BMD, and total hip T-scores were all significantly lower in those who had received recent bisphosphonate treatment as compared with those who had not.

**Table 5:** Effects of recent bisphosphonate treatment on BMD and biochemical markers of bone turnover

Variable	No recent bisphosphonate (n = 251)	Recent bisphosphonate (n = 98)	*p*-Value
Serum CTX µg/L	0.19 [0.03–0.83]	0.13 [0.01–0.63]	< 0.001
Serum PINP µg/L	35.6 [9.4–190.2]	23.1 [7.9–175.1]	< 0.001
Lumbar spine BMD (g/cm^2^)	0.856 ± 0.187	0.853 ± 0.186	0.912
Lumbar spine T-score	− 2.08 ± 1.84	− 2.28 ± 1.53	0.378
Femoral neck BMD (g/cm^2^)	0.750 ± 0.147	0.714 ± 0.145	0.070
Femoral neck T-score	− 1.37 ± 1.31	− 1.77 ± 1.07	0.016
Total hip BMD (g/cm^2^)	0.839 ± 0.137	0.794 ± 0.161	0.017
Total hip T-score	− 1.10 ± 1.22	− 1.55 ± 1.20	0.006

Values are means ± SD for BMD T-Scores and medians and range for CTX and PINP. For lumbar spine, BMD measurements were available in 93 participants who recently had been treated with bisphosphonates and 233 participants who had not. Corresponding numbers for femoral neck BMD were 79 and 209, and for total hip BMD were 79 and 208. Measurements of CTX and PINP were available on 94 participants who had been treated with bisphosphonates and 229 who had not. Reference ranges for CTX and PINP are shown in the methods section. The p-values for differences between groups for BMD T-score were calculated by t-test whereas those for CTX and PINP were calculated by Wilcoxon Test

### Influence of Pathogenic Variant Class on BMD and Biochemical Markers of Bone Turnover

We evaluated the relationship between pathogenic variant class on BMD and biochemical markers of bone turnover. The associations with biochemical markers of bone turnover are shown in Table 6. For CTX, the subgroups of individuals with no variant or VUS differed significantly from those with quantitative, qualitative, and splice site variants, whether or not they had received bisphosphonate treatment, although the effects were most marked in the untreated group. For PINP, the results were similar but, in this case, qualitative variants also differed from quantitative and splice variants in the untreated group, whereas in the treated group, those with no variant differed from the quantitative and splice site variants.

**Table 6:** Relation between class of genetic variant and biochemical markers of bone turnover

Marker	Variant type	n	No recent bisphosphonate (n = 251)	n	Recent bisphosphonate (n = 98)
CTX (ng/mL)	None and VUS	25	0.333 ± 0.023^a^	6	0.276 ± 0.047^b^
	Qualitative	74	0.239 ± 0.013	31	0.146 ± 0.021
	Quantitative	97	0.208 ± 0.012	38	0.146 ± 0.019
	Splice site	33	0.198 ± 0.020	19	0.140 ± 0.026
PINP (ng/mL)	None and VUS	25	70.7 ± 4.8^a^	6	50.4 ± 9.8
	Qualitative	74	49.8 ± 2.8^c^	31	27.4 ± 4.3
	Quantitative	97	32.8 ± 2.4	38	24.8 ± 3.9
	Splice site	33	39.5 ± 4.1	17	22.2 ± 5.5

Values are estimated marginal means and standard errors of the mean. The number of observations in each subgroup are indicated. Statistical significance between groups indicated as follows with Bonferroni adjustment for multiple comparisons: ^a^*p* < 0.001 none and VUS *vs.* quantitative and splice site, *p* = 0.002 *vs*. quantitative, ^b^*p* = 0.047 none and VUS *vs*. qualitative

^c^*p* < 0.001 qualitative *vs.* quantitative

The associations between variant type and BMD are shown in Table 7. We found that for lumbar spine BMD, qualitative variants had a significantly higher BMD than splice site variants in the previously untreated group, but there were no other significant differences between BMD and variant type at the lumbar spine, femoral neck, or total hip sites according to variant type.

**Table 7:** Relation between class of genetic variant and bone mineral density

Site	Variant type	n	No recent bisphosphonate (n = 251)	n	Recent bisphosphonate (n = 98)
Lumbar Spine BMD (g/cm^2^)	None and VUS	27	0.874 ± 0.035	7	0.968 ± 0.069
	Qualitative	71	0.812 ± 0.022	28	0.791 ± 0.034
	Quantitative	101	0. 864 ± 0.018^a^	38	0.885 ± 0.029
	Splice site	34	0.914 ± 0.031	19	0.810 ± 0.042
Femoral Neck BMD (g/cm^2^)	None and VUS	25	0.791 ± 0.029	7	0.770 ± 0.058
	Qualitative	50	0.747 ± 0.021	22	0.695 ± 0.031
	Quantitative	101	0.737 ± 0.015	34	0.728 ± 0.025
	Splice site	33	0.764 ± 0.025	15	0.670 ± 0.038
Total Hip BMD (g/cm^2^)	None and VUS	25	0.890 ± 0.028	7	0.843 ± 0.054
	Qualitative	50	0.847 ± 0.020	22	0.777 ± 0.030
	Quantitative	100	0.819 ± 0.014	34	0.748 ± 0.024
	Splice site	33	0.849 ± 0.025	15	0.788 ± 0.037

Values are estimated marginal means and standard errors of the mean. The number of observations in each subgroup are indicated. Statistical significance between groups indicated as follows with Bonferroni adjustment for multiple comparisons: ^a^*p* = 0.046, qualitative *vs.* splice site. Abbreviations: *BMD* Bone Mineral Density in g/cm^2^; *VUS* Variant of uncertain significance

## Discussion

Osteogenesis imperfecta is a heterogeneous disease that affects many organ systems, which is characterised by a greatly increased risk of low-energy fractures [1]. These fractures peak during childhood and gradually decrease as the skeleton grows, but the incidence of fractures is much higher in OI throughout life as compared with population-based controls [2].

The pathogenesis of OI-related fractures is incompletely understood. Previous studies in adults have reported that BMD values are lower in OI than in controls [16] or in comparison with the normal reference range [17] but reductions in BMD cannot explain the marked increase in fracture risk that is observed in OI. Reflecting this fact, Wekre and colleagues reported that only 10% of people with OI had BMD values in the osteoporotic range [18], but they also commented that the rate of fracture in this subgroup was three times higher than those with non-osteoporotic T-scores. However, in this publication, no detail was provided on what the fracture rates actually were in these subgroups. In the same study, total body BMD and BMC were significantly associated with fracture numbers, but no further detail was provided. In the TOPaZ study, we found that the proportion of individuals with osteoporosis was higher than that reported by Wekre [18], but that this differed according to the site of measurement. At the lumbar spine, T-scores were in the osteoporotic range in 157 subjects (45.0%), whereas corresponding values for the femoral neck were 65 (18.6%) and for the total hip 39 (11.1%). In contrast to the Wekre study [18], we found no significant association between fracture numbers in the two years prior to enrollment in subjects with osteoporosis, osteopenia, and normal BMD. This is most probably due to the fact that fracture risk in OI is not only influenced by bone density, but also by reduced bone quality as the result of abnormalities of type I collagen structure and mineralisation [4].

The genetic causes of OI in TOPaZ were consistent with those in previous cohort studies of OI. A study in the Swedish OI population reported by Lindahl [19] reported that pathogenic variants in the *COL1A1* and *COL1A2* genes were the most common causes of OI and were found in 85.9% of individuals. Similarly, a study of 85 individuals with OI in the Danish population reported the presence of pathogenic variants in *COL1A1* or *COL1A2* in 81 cases (95%), but in 4 individuals (5%), no pathogenic variants were detected in genes known to cause OI. In the TOPaZ study, pathogenic variants in *COL1A1* or *COL1A2* were found in 85%, but other implicated genes included *FKBP10, IFITM5, P3H1*, and *BMP1*. Interestingly, no pathogenic variants could be detected in genes in 31 individuals (8.9%) despite the fact they had been diagnosed clinically with OI. It is possible these individuals may have had another cause for bone fragility or may have harboured pathogenic variants in novel genes that cause OI.

Only few studies have investigated the relation between genotype and skeletal phenotype in OI. The Lindahl study [20] reported significantly higher BMD (expressed as Z-score) in Type I versus both type III and IV but this work was not directly comparable to our study as it comprised a mixture of adults and children. In the same study, no differences were found in BMD Z-score according to whether pathogenic variants were qualitative or quantitative. Hald and colleagues previously reported that lumbar spine BMD in type I OI was significantly higher than types III and IV and that a similar pattern was observed for the femoral neck and total hip [21]. Similar findings have been reported by Ohata and colleagues in 53 people with OI from Japan [22]. These previous reports are in keeping with the TOPaZ study where BMD values were significantly higher with type I than the other classical OI types. Interestingly, those with an unknown type had higher BMD values than type III at the lumbar spine and types III and IV at the femoral neck.

We also evaluated associations between recent bisphosphonate treatment and both biochemical markers of bone turnover and BMD. We found that individuals who had received bisphosphonates during the 2 years prior to recruitment had lower CTX and PINP values than those who did not – a result that is expected given the known inhibitory effects of bisphosphonates on bone turnover. We also found that those who had received bisphosphonates during the prior 2 years had significantly lower BMD at the femoral neck and total hip than those who had not received bisphosphonates. Although this is at first sight surprising, we speculate that it may have been due to the fact that clinicians are more likely to prescribe bisphosphonates for individuals with low BMD in routine clinical practice.

We also evaluated associations between the class of genetic variant and BMD but found no significant differences between variant subtypes and BMD except at the lumbar spine where qualitative variants had higher BMD than those with quantitative variants.

Biochemical markers of bone turnover have been little studied in adults with OI. In the Orwoll clinical trial [11], baseline PINP values and the marker of bone resorption, urinary NTx creatinine values, were within the normally accepted reference ranges; the average ± SD PINP was 34.7 ± 25.0 µg/L and NTx/creatinine was 50.6 ± 38.5 nM BCE/mM (typical reference range 17–125). Similarly, Adami et al. reported that baseline biochemical bone markers, serum bone alkaline phosphatase, and serum CTX, were within the normal range in a study including adults with OI type I, III, and IV [23]. In Hald’s study, no significant difference was observed between biochemical markers and either Sillence type or class of genetic variant [21]. The observations in TOPaZ are in keeping with these previous studies, as we found that average values for the biochemical markers of bone turnover CTX and PINP were generally within the reference range. Subgroup analysis revealed that those with no pathogenic variant and variants of uncertain significance had significantly higher CTX and PINP values than those who carried other variant types. The difference was most pronounced in those who had not recently been treated with bisphosphonates. We also found that PINP values were significantly higher in those with qualitative variants compared with quantitative in those who had not been treated with bisphosphonates recently.

The overall aim of TOPaZ is to determine if the combination of TPTD followed by the antiresorptive drug zoledronic acid is effective at reducing the risk of clinical fractures in adult OI compared with standard care. The reason for choosing TPTD was based on the study by Orwoll, who reported encouraging results on BMD in adult patients with osteogenesis imperfecta when TPTD was compared with placebo, but this study was small and was not designed to look at fracture prevention [11]. The reason for using ZA following TPTD in TOPaZ was to maintain any increases in BMD that may occur as a result of anabolic therapy. There have been various other randomised trials and observational studies of bisphosphonates in both children and adults with osteogenesis imperfecta [9]. A Cochrane review concluded that while there was good evidence that bisphosphonates increase BMD in OI, there was no convincing effect on fracture reduction [10].

In the absence of a licensed medical treatment for fracture prevention in OI, bisphosphonates are widely used on an empirical basis in the treatment of OI in both children and adults [10]. Reflecting this fact, 28.1% of individuals in our cohort had received treatment with a bisphosphonate in the 2 years prior to enrolment and 70.2% had used bisphosphonates more than 2 years prior to enrolment. Other drugs were used much less commonly but included teriparatide, denosumab, strontium ranelate, raloxifene, HRT, and calcitonin.

There are limitations to the data reported. Information on previous fractures may have been underestimated as the result of recall bias. This is particularly likely to be the case for childhood fractures. Furthermore, health records only report fractures seen in the hospital and documented by radiographs, which is not always the case in adults with OI. Additionally, BMD measurements were frequently unavailable, particularly in patients with type III and IV OI where metalwork and image artefacts associated with previous fractures prevented us from assessing BMD. Although many participants were at the age where Z-scores rather than T-scores are recommended by the International Society for Clinical Densitometry as the preferred means of expressing BMD, we elected to use T-scores for consistency and so that a comparison could be made across different subtypes of OI. On the other hand, a major strength of the study is the large sample size, which makes the study cohort one of the largest ever assembled in adults with OI. Whatever the eventual outcome of the TOPaZ trial, the cohort represents a valuable resource with which to investigate the clinical characteristics of adults with OI and genotype–phenotype relationships regarding associations between molecular diagnosis, bone density, biochemical markers of bone turnover, and other non-skeletal comorbidities. When the trial reports, it will provide new insights into the fracture rate in adults with OI and the effect of an anabolic therapy followed by antiresorptive treatment in the management of this condition.
